# Sub-retinal abscess as presenting feature of endogenous *Candida* endophthalmitis

**DOI:** 10.1186/s13104-018-3682-1

**Published:** 2018-08-17

**Authors:** Sidra Zafar, M. A. Rehman Siddiqui

**Affiliations:** 1 Section of Ophthalmology, Aga Khan University Hospital, Stadium Road Karachi, Karachi, Pakistan; 2 Section of Ophthalmology, South City Hospital, Karachi, Pakistan

**Keywords:** Candida, Fungal, Endophthalmitis, Subretinal abscess

## Abstract

**Background:**

Sub-retinal abscess as the presenting feature in the setting of endogenous fungal endophthalmitis is extremely infrequent. Immunodeficiency states are major predisposing risk factors. To the best of our knowledge, this is the first case report of *Candida* sub-retinal abscess as initial presentation in an immunocompetent patient.

**Case presentation:**

A 32-year old, generally fit and well, female presented to us with gradually deteriorating vision in her right eye. Visual acuity was counting fingers in the right eye and, 20/30 in the left eye. Right eye fundus examination showed a full thickness, yellowish-white foveal lesion, and significant vitreous haze. Left eye examination was normal. Upon direct questioning, the patient disclosed history of backstreet abortion 3 weeks prior to the onset of her ocular symptoms. She underwent vitreous tap and intravitreal antibiotics (amphotericin B, 5 μg/0.5 ml). Vitreous culture showed profuse growth of *Candida albicans*. Because her condition was progressively deteriorating, she underwent 25 g vitrectomy plus repeat intravitreal amphotericin B under general anaesthesia. Three weeks post-vitrectomy, vitreous inflammation resolved completely, and the sub-retinal abscess healed with a macular scar formation. Over a follow-up of 4 years, no recurrences were observed.

**Conclusion:**

Our case highlights the importance of considering *Candida albicans* infection in the differential diagnosis of sub-retinal abscesses. Although immunocompromised states are traditionally identified as predisposing factors for fungal infections, fungal endogenous endophthalmitis can occur in healthy individuals as well.

## Background

Endogenous endophthalmitis is an uncommon clinical condition accounting for approximately 2–8% of all endophthalmitis cases [1]. Sub-retinal abscess as the predominant presentation of severe endophthalmitis is even rarer. Here, we present a case of endogenous *Candida* endophthalmitis complicated by sub-retinal abscess following surgical abortion in an immunocompetent woman.

## Case presentation

A 32-year old female presented to us with progressively deteriorating vision in her right eye for 2 months.

**Fig. 1:**
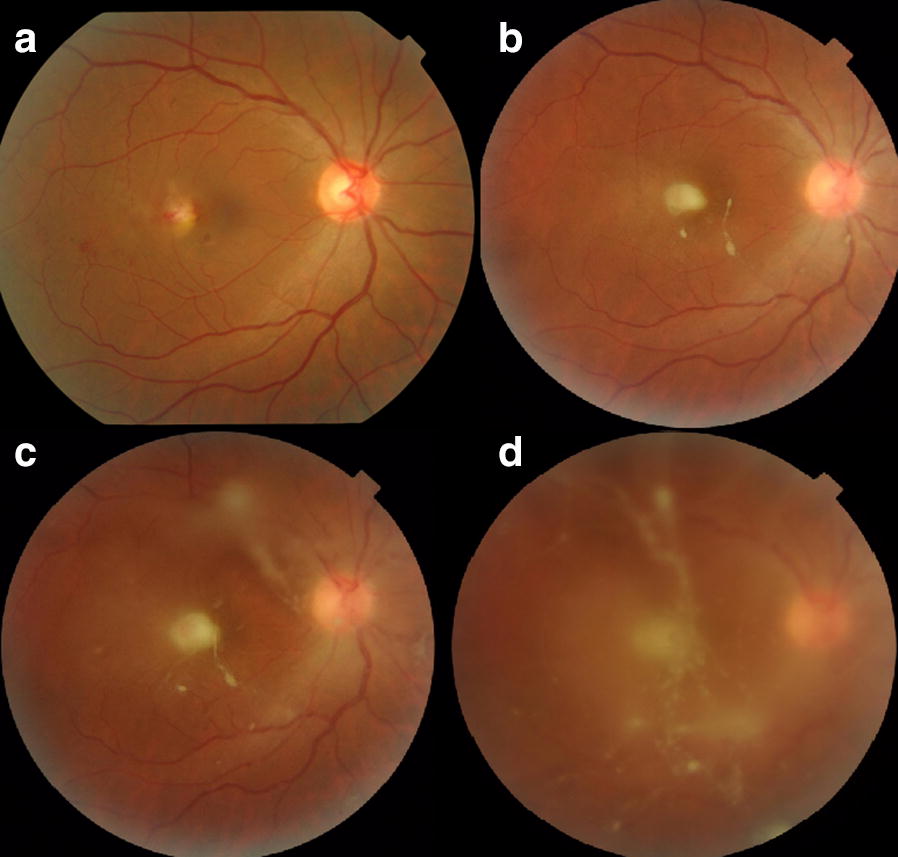
**a**–**d** Right eye fundus photographs showing presence of a sub-retinal abscess, with vitreous clumps resembling ‘pearls on a string’. **a** At presentation to outside ophthalmologist, **b** upon presentation to us, and **c**, **d** progressive worsening even with intravitreal anti-fungal therapy

When the patient presented to us, visual acuity (VA) in her right eye was counting fingers and in the left eye, it was 20/30. Anterior segment examination was within normal limits for both eyes and intraocular pressures were 14 mmHg. Right eye fundus examination was significant for vitritis, with vitreous clumps manifesting as classic ‘pearls on a string’ appearance. A full thickness, yellowish-white foveal lesion was also appreciated (Fig. 1). Left eye examination was normal. Uveitis workup was unremarkable, except for an elevated erythrocyte sedimentation rate (ESR = 38). Mantoux test (0 mm), VDRL, FT-ABS, ANA, ASMA, AMA, and Toxoplasma IgG and IgM were all negative. Prior to consulting us, she had seen outside ophthalmologists where she was given intravitreal triamcinolone acetate in the affected eye. In addition, systemic prednisolone therapy had also been given. However, no improvement was observed with these treatments. A diagnosis of Toxoplasma retinochoroiditis was presumed and the patient was started on empiric Septran DS (sulfamethoxazole and trimethoprim).

On direct questioning, the patient revealed having undergone back-alley abortion 3 weeks prior to onset of her ocular symptoms, and that she was also suffering from vaginal discharge. She was referred to gynaecology, who felt, that the two problems were unrelated. Vaginal swabs were however taken, and Gram-positive rods were identified on Gram staining. A diagnosis of bacterial vaginosis was made, and she was commenced on Metronidazole.

**Fig. 2:**
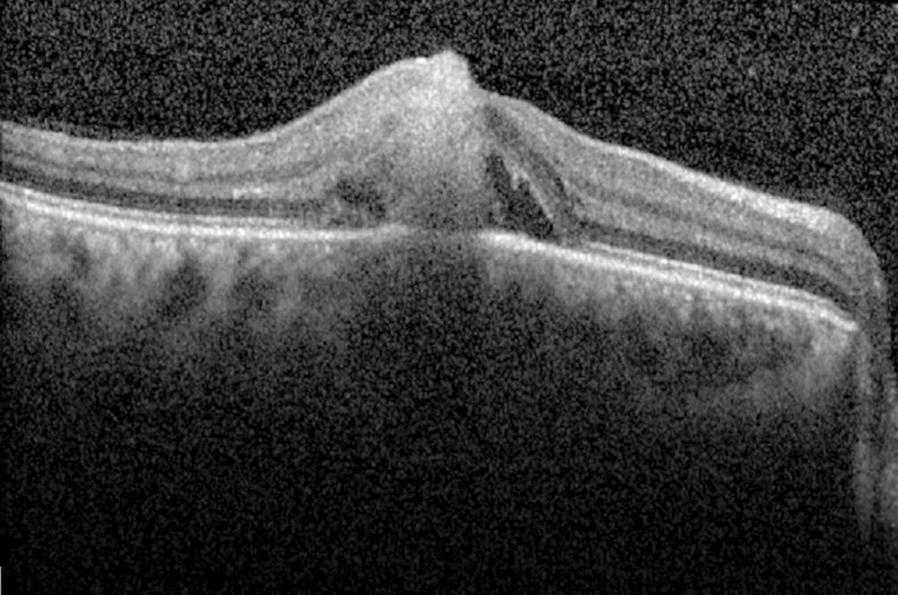
Right eye OCT image depicts a sub-foveal conical lesion extending from the choroid into the full thickness of retina, corresponding to *Candida* sub-retinal abscess

**Fig. 3:**
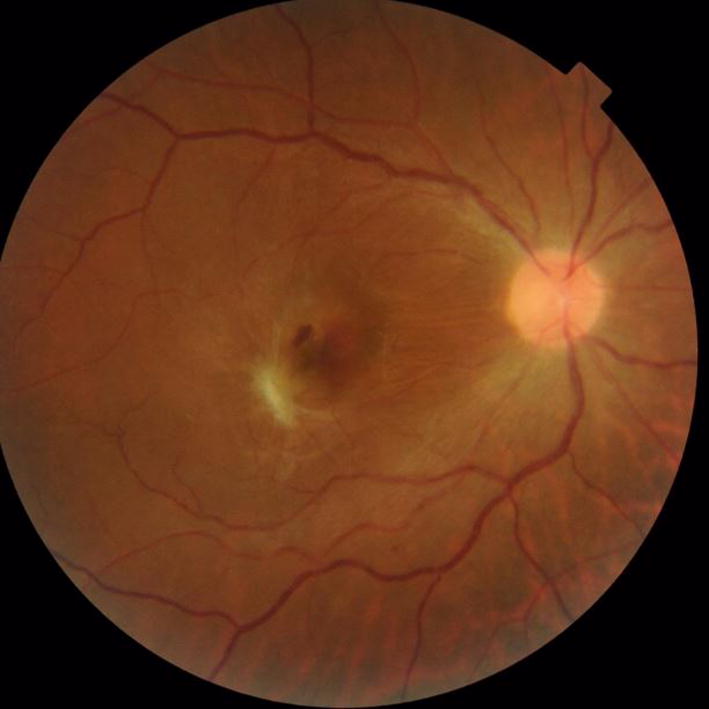
Status post-vitrectomy fundus photograph. Inflammation has resolved and a macular scar can be appreciated

Based on the patient’s history and clinical findings, we strongly suspected a fungal sub-retinal abscess. Retinal imaging, which included optical coherence tomography (OCT) and fundus fluorescein angiography (FFA), were ordered. OCT of the right eye showed a sub-foveal conical lesion extending from the choroid into the full thickness of retina (Fig. 2). The apex of the lesion was directed towards the inner retina and formed a tiny protuberance (roof of the abscess). Blood cultures were requested but did not reveal any microbial growth. A vitreous tap was subsequently performed, and intravitreal amphotericin B 5 μg/0.5 ml was administered. Vitreous culture showed profuse *Candida albicans* growth. Because of limited response to intravitreal antibiotics, therapeutic 25 g pars plana vitrectomy, with gas tamponade and repeat intra-vitreal amphotericin B was performed. She was also commenced on oral voriconazole. The eye responded well to debulking surgery. Three weeks post-vitrectomy, inflammation resolved completely with macular scar formation (Fig. 3). During the subsequent 4 years follow-up, the patient had no recurrences.

## Discussion and conclusion

Endogenous *Candida* endophthalmitis is a potentially devastating ocular condition. Visual outcomes are often poor and incidence rates vary around 9.9–45% in individuals with underlying candidemia. Predisposing risk factors include intravenous drug abuse, immunodeficiency states, and prolonged systemic corticosteroid or antibiotic therapy [2–4]. Albeit very rare, cases of *Candida* endophthalmitis have also been described in young immunocompetent women either during pregnancy or in the post-partum period. Presence of intrauterine devices, *Candida* vaginitis, antibiotic/steroid therapy and invasive procedures, including surgical abortion are identified as possible predisposing factors for pregnancy-associated *Candida* sepsis [5–10]. Once in the bloodstream, *Candida* gains access to the eyes via the short posterior ciliary artery. Infection typically progresses, vertically, via chorioretinal infiltration and vitreous is a primary site of localisation. It is suggested that, the higher glucose concentration in vitreous supports the growth of *Candida* [11]. In contrast, if the infection assumes a horizontal course spreading under the sensory retina or retinal pigment epithelium, a sub-retinal abscess may result [12, 13]. To the best of our knowledge, sub-retinal abscess formation with endogenous *Candida* endophthalmitis has only been reported twice in literature [13, 14]. Additionally, the lesion was not observed in any of the obstetric cases that were complicated by Candidemia and endophthalmitis.

Sub-retinal abscess is an extremely uncommon manifestation of endogenous endophthalmitis. The abscess typically appears as a solitary, yellowish-white mass-like elevation. Additional accompanying features may include retinal haemorrhages and cellular vitreous reaction. Visual prognosis is often poor and because of disease rarity, there are no definite guidelines for sub-retinal abscess treatment. Both bacterial and fungal etiologies have been implicated, with bacterial etiology being more common [12–14].

Although *Candida* is the most common fungal cause of endogenous endophthalmitis, only two cases of *Candida*-associated sub-retinal abscess have been described to date. Kaburaki et el [14] reported a case of *Candida albicans* endophthalmitis complicated by sub-retinal abscess formation in a liver transplant patient. Arai et al. described a similar case but with bilateral presentation in a patient on high-dose systemic corticosteroids for interstitial pneumonia and with underlying rheumatoid arthritis [13]. In comparison, our patient was immunocompetent, and had an unremarkable past medical history, barring a surgical abortion. We believe that, use of oral prednisolone even for a short duration, may have predisposed our patient to developing a more severe and aggressive disease course. Chen et al. for instance, reported *Candida* endophthalmitis in two healthy females following surgical abortion. Despite both women receiving the same treatment, one of them had a more severe clinical course. The patient in this scenario had received prior systemic corticosteroid therapy; her disease course was complicated by recurrent retinal detachments and final visual acuity of counting fingers [6].

Both Kaburaki et al. and Arai et al. reported difficulty in isolating the causative microorganism using vitreous fluid specimens. Kaburaki et al. performed histopathological examination of epiretinal proliferative tissue for diagnostic confirmation [14], whereas Arai et al. performed repeated vitreous taps and polymerase chain reaction (PCR) assays [13]. In the present case, we were able to successfully isolate *Candida albicans* twice; once on initial vitreous tap, and then from vitrectomy fluid sample. In a 20-year review of fungal endophthalmitis cases, vitrectomy yielded positive results in 92% of eyes when used as the initial diagnostic procedure. In comparison, anterior chamber paracentesis and vitreous tap without vitrectomy yielded positive culture results in 25% and 44% of eyes, respectively [15].

*Candida* associated inflammation often manifests as formation of multiple micro abscesses in the vitreous which necessitates vitrectomy for complete resolution of infection [11]. However, despite multi-modal management, final visual acuity is often sub-optimal. A recently published study evaluated OCT changes in patients with established *Candida* endogenous endophthalmitis. They reported that in eyes with a chorioretinal pattern of *Candida* involvement, extension of the microorganism beyond inner nuclear layers was highly associated with scar formation and relatively poor visual recovery [16].

Our case highlights the importance of including *Candida albicans* infection in the differential diagnosis of sub-retinal abscesses, even in the immunocompetent. The correct diagnosis for our patient was made after a delay of almost 2 months requiring multiple visits. Ophthalmologists should also be more careful when initiating corticosteroids until infectious etiology has been completely ruled out.

## Abbreviations

FFA: fundus fluorescein angiography
I/V: intravitreal
OCT: optical coherence tomography
